# Prevalence and epidemiological pattern of drug-resistant tuberculosis among migrant populations in Wenzhou City, China, 2014–2023: implications for public health strategies

**DOI:** 10.3389/fpubh.2025.1600214

**Published:** 2025-08-15

**Authors:** Lianpeng Wu, Xiyue Cai, Shuya Xu, Xuefeng Lin, Shuangliao Wu, Xueqin Xu

**Affiliations:** ^1^Department of Clinical Laboratory Medicine, The Ding Li Clinical College of Wenzhou Medical University, Wenzhou Central Hospital, Wenzhou, China; ^2^Key Laboratory of Diagnosis and Treatment of New and Recurrent Infectious Diseases of Wenzhou, Wenzhou Sixth People's Hospital, Wenzhou, China; ^3^Medical Management Office, Wenzhou Municipal Public Hospital Management Center, Wenzhou, China; ^4^Department of Tuberculosis Clinic, The Ding Li Clinical College of Wenzhou Medical University, Wenzhou Central Hospital, Wenzhou, China; ^5^Department of Clinical Laboratory Medicine, Yueqing People's Hospital, Wenzhou, Zhejiang, China; ^6^Key Laboratory of Precision Medicine of Wenzhou, Wenzhou Central Hospital, Wenzhou, China

**Keywords:** multidrug-resistant tuberculosis, epidemiology, notified cases, migrant population, Wenzhou

## Abstract

**Objective:**

This study aimed to analyze the epidemiological characteristics and trends of notified multidrug-resistant tuberculosis (MDR-TB) in Wenzhou City, China, from 2014 to 2023, with a focus on differences between migrant and local populations among reported TB cases.

**Methods:**

This was a facility-based retrospective cohort study that included all bacteriologically confirmed TB cases notified between 1 January 2014 and 31 December 2023 in the Tuberculosis Information Management System (TBIMS) of the Chinese Center for Disease Control and Prevention and the hospital’s laboratory information system, provided they had available phenotypic drug-susceptibility testing (pDST) results. Pearson’s chi-square test was used to compare drug-resistance rates between groups, the trend chi-square test was applied to assess temporal changes, and a Sankey diagram was employed to illustrate the origins and intra-city distribution of MDR-TB among the migrant population.

**Results:**

Among 10,993 notified TB patients, 734 (6.68%) were classified as MDR-TB. The proportion of MDR-TB among notified cases declined over the study period (*p* < 0.001). Nearly half (352/734; 47.96%) of the notified MDR-TB patients were migrants; 226 (64.21%) originated from elsewhere in Zhejiang Province, and 126 (35.79%) came from outside the province. Guizhou, Jiangxi and Sichuan were the leading external contributors. Within Wenzhou, Yueqing City, Yongjia County and Ouhai District reported the highest numbers of migrant MDR-TB notifications.

**Conclusion:**

The proportion of MDR-TB among notified TB cases in Wenzhou City has steadily decreased. Migrants account for almost half of these notified MDR-TB cases. Surveillance-driven and migrant-targeted interventions should be prioritized to further reduce MDR-TB transmission.

## 1 Introduction

Tuberculosis (TB), a persistent global health threat (1), caused an estimated 1.25 million deaths in 2023, surpassing COVID-19 as the leading infectious disease killer (1, 2). While global TB incidence rates declined by 2% annually from 2010 to 2020, recent years have seen a resurgence, with 10.8 million new cases projected for 2023—a 4.6% increase from 2020 levels (3). Drug-resistant TB (DR-TB), particularly multidrug-resistant TB (MDR-TB), compounds this crisis. In 2023, 400,000 new MDR/rifampicin-resistant TB (RR-TB) cases emerged globally, yet only 44% were diagnosed and treated, with treatment success rates stagnating below 70% (3). MDR-TB’s prolonged treatment duration, high costs, and socioeconomic burden underscore the urgent need for targeted interventions (4, 5).

In recent years, Wenzhou City has implemented several measures to control MDR-TB, including expanding phenotypic drug sensitivity testing (pDST) to most bacteriologically positive patients, equipping all designated TB hospitals with the GeneXpert MTB/RIF system for rapid rifampicin resistance detection, incorporating MDR pulmonary TB into medical insurance with subsidies up to 30,000 yuan per patient, and increasing financial support for designated TB hospitals. These efforts have improved local MDR-TB control, but challenges remain in providing TB services to vulnerable populations, such as migrants (6).

In the context of rapid social and economic development in China, population movements have become increasingly frequent (7). As an economically developed coastal city, Wenzhou has attracted a significant number of migrants. The living conditions of this migrant population are often complex, characterized by overcrowding and inadequate sanitation facilities, which facilitate the spread of MDR-TB (8). Additionally, previous studies (9, 10) have indicated that the frequent relocation of migrants can lead to challenges in accessing medical care across different regions, resulting in interrupted treatment and irregular medication adherence. This situation increases the likelihood of developing MDR-TB among the migrant population. Understanding the epidemiological characteristics and trends of MDR-TB within this group is therefore essential for the timely detection and management of mobile patients and for effective epidemic control. To date, such evidence has been lacking for Wenzhou. This study aims to analyze the epidemiological features and temporal trends of MDR-TB among notified TB cases in Wenzhou from 2014 to 2023 and to compare patterns between migrant and local populations, so as to inform the development of targeted prevention and control strategies.

## 2 Materials and methods

### 2.1 Study setting, population, and data collection

**Figure 1:**
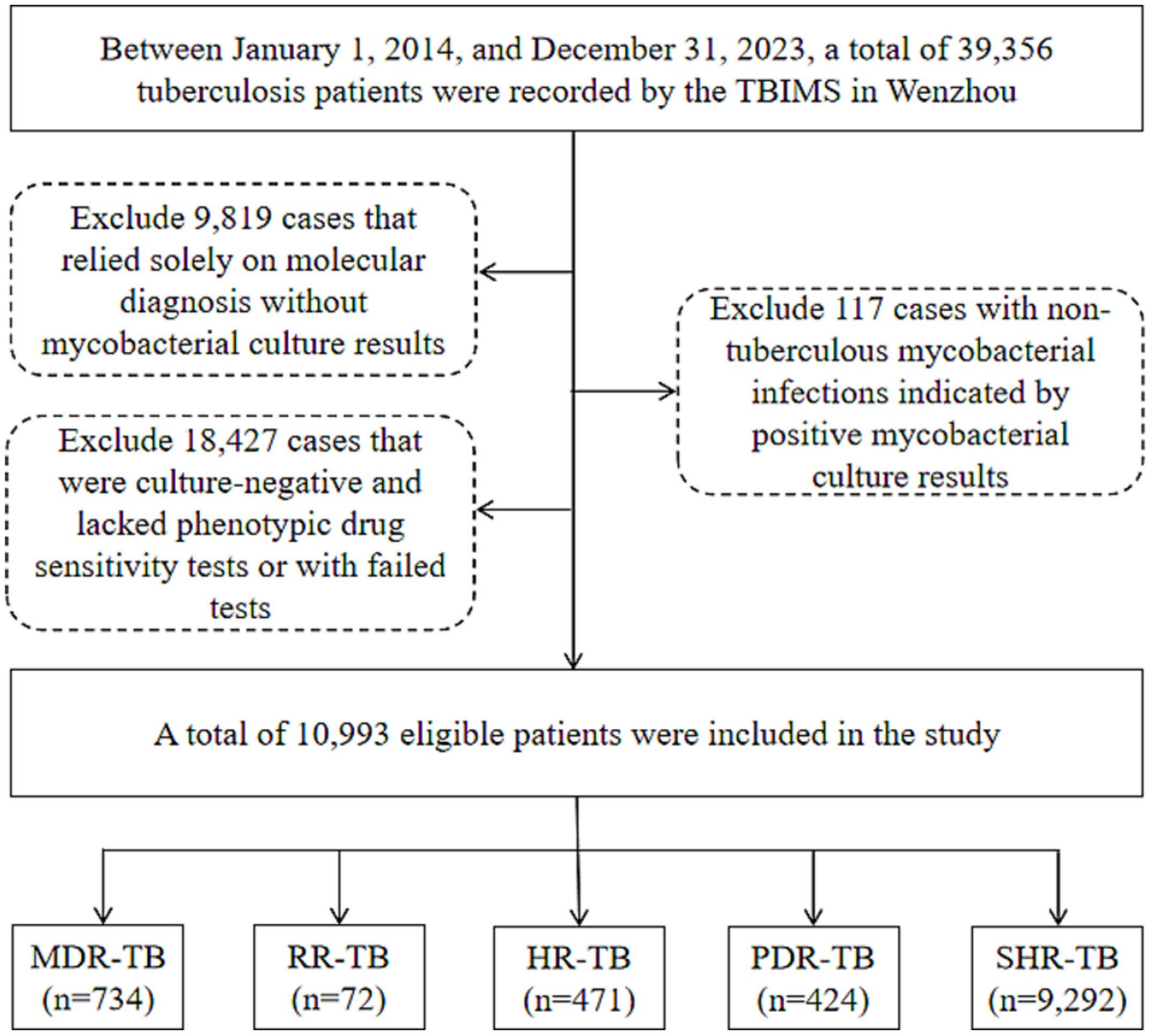
Flow chart of the patients enrolled in the study. TBIMS, tuberculosis information management system; MDR-TB, multidrug-resistant tuberculosis; RR-TB, rifampicin-resistant tuberculosis; HR-TB, isoniazid-resistant tuberculosis; PDR-TB, polydrug-resistant tuberculosis; SHR-TB, isoniazid rifampicin-sensitive tuberculosis.

The research was carried out in Wenzhou, a city located on China’s southeast coast. Wenzhou’s geographical coordinates extend from 119° 37′ to 121° 18′ east longitude and from 27° 03′ to 28° 36′ north latitude. Covering an area of 12,110 square kilometers, the city is made up of four districts, five counties, and three county-level cities. As of late 2023, the permanent population of Wenzhou is around 9.761 million, which includes a transient population of 4.42 million. This research took place at Wenzhou Central Hospital, the sole facility in Wenzhou dedicated to treating drug-resistant tuberculosis. The sociodemographic information (including gender, age, ethnicity, household registration address and category [local or migrant], current residence, and occupation), phenotypic drug susceptibility testing (pDST) outcomes, and the tuberculosis treatment history of participants were sourced from the Tuberculosis Information Management System (TBIMS) of the Chinese Center for Disease Control and Prevention and the hospital’s laboratory information system. Between January 1, 2014, and December 31, 2023, the TBIMS recorded a total of 39,356 patients diagnosed with tuberculosis in Wenzhou. Of these, 9,819 cases that depended exclusively on molecular diagnosis without results from mycobacterial cultures were removed from consideration. Furthermore, 117 cases that presented nontuberculous mycobacterial infections as indicated by positive mycobacterial culture results were also excluded, along with 18,427 cases that were culture-negative and lacked pDST results or having failed such tests. In total, 10,993 patients who met the eligibility criteria were incorporated into the study. The specifics concerning the patient inclusion and exclusion criteria are depicted in Figure 1.

### 2.2 Definitions

The migrant population refers to people whose place of residence is inconsistent with their registered household location at the township or street level, and who have been away from their registered household location for six months or more. Multidrug-resistant tuberculosis (MDR-TB) is defined as resistance to at least both isoniazid and rifampicin (11). Polydrug-resistant tuberculosis (PDR-TB) is defined as resistance to two or more first-line anti-TB drugs (except both isoniazid and rifampicin) (11). Rifampicin-resistant tuberculosis (RR-TB) is defined as resistance to rifampicin only. Isoniazid-resistant tuberculosis (HR-TB) is defined as resistance to isoniazid only. Isoniazid rifampicin-sensitive tuberculosis (SHR-TB) is defined as susceptibility to both isoniazid and rifampicin.

### 2.3 Bacteriologic examinations and drug susceptibility testing

Upon hospital admission, 3–5 mL sputum or bronchoalveolar lavage fluid samples were rigorously collected and pretreated with NALC-NaOH solution. This pretreatment step is critical for sample decontamination and significantly enhances the isolation rate of potential pathogens. The processed samples were then inoculated into BACTEC MGIT liquid culture tubes (BD, USA) or Löwenstein-Jensen solid media (Zhuhai BaiShi Biotechnology Co., Ltd.) and incubated at a constant temperature of 37°C to facilitate mycobacterial growth. For culture-positive isolates, smears were prepared and subjected to Ziehl-Neelsen acid-fast staining (KS, Seoul, Korea). This staining technique is definitive for mycobacterial identification by differentiating acid-fast bacilli from other bacteria under microscopic examination. When mycobacteria were detected, MPB64 antigen testing (Hangzhou Innovation Biotechnology Co., Ltd.) was further performed to confirm the identification of *Mycobacterium tuberculosis* (MTB), the causative agent of tuberculosis. In addition to strain identification, phenotypic drug susceptibility testing (pDST) for MTB was rigorously conducted using the MGIT liquid method. The procedure strictly adhered to the manufacturer’s protocols for both the instrument and reagents to ensure result reliability. The pDST evaluated the inhibitory effects of four first-line anti-tuberculosis drugs at the following concentrations: streptomycin (SM) 1.0 μg/mL, isoniazid (INH) 0.1 μg/mL, rifampicin (RFP) 1.0 μg/mL, and ethambutol (EMB) 5.0 μg/mL.

### 2.4 Statistical analysis

All data were imported into WPS Excel (version 12.1.0.18276, Jinshan Office Software Co., Ltd., Beijing, China) to create a database. Additionally, WPS Excel was utilized for generating trend charts related to drug resistance. Statistical analyses were carried out with SPSS software (version 26.0, IBM, New York, USA), where counting data were represented in terms of frequencies and percentages. To compare categorical variables, Pearson’s chi-square test was applied. The trend chi-square test was used to examine the temporal patterns associated with TB drug resistance. Furthermore, Origin software (version 2021, OriginLab, Massachusetts, USA) assisted in developing a Sankey diagram, depicting the sources and destinations of migrant MDR-TB. A significance level of *p*-value < 0.05 was deemed statistically significant.

## 3 Results

### 3.1 Demographic characteristics of the study population

A total of 10,993 subjects were included in this study, with a median age of 45 years (29, 59). Among these, 7,930 were males (72.14%) and 3,063 were females (27.86%). The majority of participants were of Han nationality, comprising 10,681 cases (97.16%), while 312 cases (2.84%) were from other ethnic minorities. Additionally, 4,786 cases (43.54%) were classified as part of the migrant population, and 6,207 cases (56.46%) were local residents. Employment status revealed that 2,965 cases (26.97%) were employed, whereas 8,028 cases (73.03%) were unemployed. Furthermore, 1,176 cases (10.70%) had previously received anti-TB treatment, while 9,817 cases (89.30%) were newly diagnosed.

### 3.2 Proportion and trends of drug-resistant TB among notified cases

**Figure 2:**
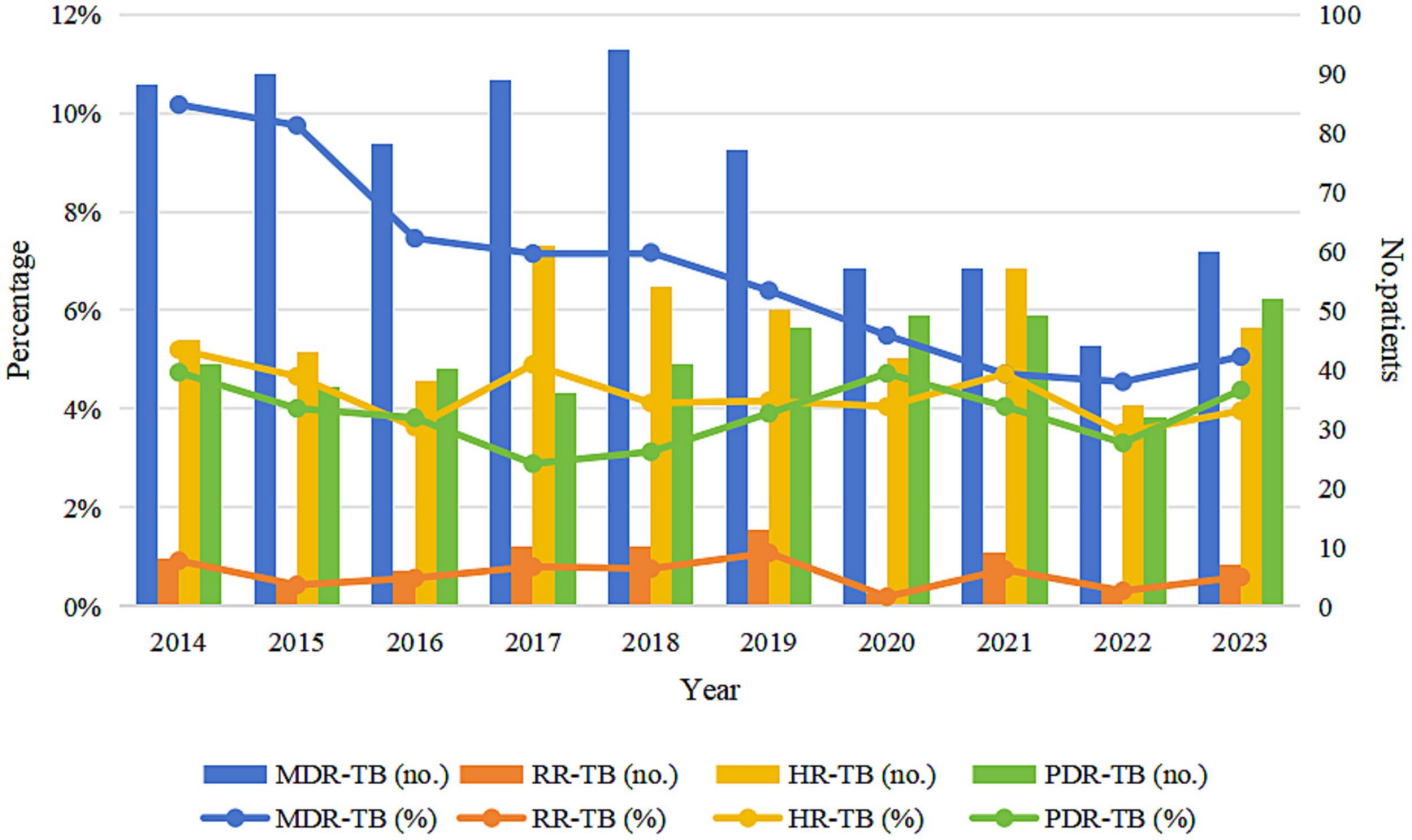
Trends in drug resistance patterns in Wenzhou from 2014 to 2023. MDR-TB, multidrug-resistant tuberculosis; RR-TB, rifampicin-resistant tuberculosis; HR-TB, isoniazid-resistant tuberculosis; PDR-TB, polydrug-resistant tuberculosis.

Among 10,993 bacteriologically-confirmed TB cases notified in Wenzhou, 734 (6.68%) were classified as MDR-TB, 72 (0.66%) as RR-TB, 471 (4.28%) as HR-TB, 424 (3.86%) as PDR-TB, and 9,292 (84.52%) as SHR-TB. From 2014 to 2023, the proportion of MDR among all notified TB cases declined significantly (*p* < 0.001), while the proportions of RR-TB, HR-TB, and PDR-TB showed modest decreases (*p* > 0.05) (Table 1; Figure 2). Among newly notified patients, 434 (4.42%) were MDR; among previously treated patients, 300 (25.51%) were MDR. The difference in the proportion of MDR between the two groups was statistically significant (χ^2^ = 749.593, *p* < 0.001).

**Table 1:** Drug resistance patterns of tuberculosis patients in Wenzhou from 2014 to 2023.

Year	No. of patients	MDR-TB (*n*, %)	RR-TB (*n*, %)	HR-TB (*n*, %)	PDR-TB (*n*, %)	SHR-TB (*n*, %)
2014	865	88 (10.17%)	8 (0.92%)	45 (5.20%)	41 (4.74%)	683 (78.96%)
2015	923	90 (9.75%)	4 (0.43%)	43 (4.66%)	37 (4.01%)	749 (81.15%)
2016	1,046	78 (7.46%)	6 (0.57%)	38 (3.63%)	40 (3.82%)	884 (84.51%)
2017	1,245	89 (7.15%)	10 (0.80%)	61 (4.90%)	36 (2.89%)	1,049 (84.26%)
2018	1,311	94 (7.17%)	10 (0.76%)	54 (4.12%)	41 (3.13%)	1,112 (84.82%)
2019	1,203	77 (6.40%)	13 (1.08%)	50 (4.16%)	47 (3.91%)	1,016 (84.46%)
2020	1,038	57 (5.49%)	2 (0.19%)	42 (4.05%)	49 (4.72%)	888 (85.55%)
2021	1,210	57 (4.71%)	9 (0.74%)	57 (4.71%)	49 (4.05%)	1,038 (85.79%)
2022	966	44 (4.55%)	3 (0.31%)	34 (3.52%)	32 (3.31%)	853 (88.30%)
2023	1,186	60 (5.06%)	7 (0.59%)	47 (3.96%)	52 (4.38%)	1,020 (86.00%)
Total	10,993	734 (6.68)	72 (0.66%)	471 (4.28%)	424 (3.86%)	9,292 (84.52%)
χ^2^		48.652	0.862	1.661	0.060	
*P*-value		< 0.001	0.353	0.197	0.806	

No. of patients, number of bacteriologically-confirmed TB cases with pDST results. MDR-TB, multidrug-resistant tuberculosis; RR-TB, rifampicin-resistant tuberculosis; HR-TB, isoniazid-resistant tuberculosis; PDR-TB, polydrug-resistant tuberculosis; SHR-TB, isoniazid rifampicin-sensitive tuberculosis.

### 3.3 Trends of MDR-TB proportion among migrant and local notified cases

**Figure 3:**
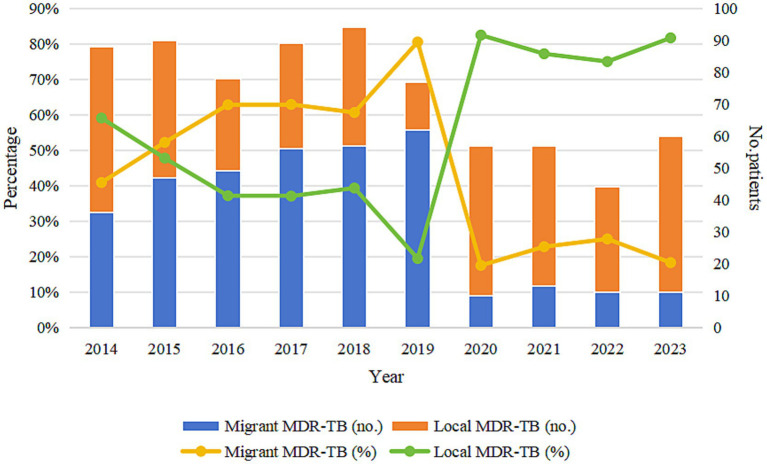
Trends in the proportion of migrant MDR-TB and local MDR-TB in Wenzhou from 2014 to 2023. MDR-TB, multidrug-resistant tuberculosis.

Among the 734 MDR-TB cases, 352 (47.96%) were notified among migrants and 382 (52.04%) among local residents. The proportion of MDR among notified migrant TB cases was 7.35%, higher than that among local notified cases (6.15%) (χ^2^ = 6.250, *p* < 0.05) (Table 2). The annual share of migrant MDR-TB cases among all MDR-TB cases rose from 2014 to 2019 and then decreased markedly after 2020 (*p* < 0.001) (Table 3; Figure 3).

**Table 2:** Proportion of MDR-TB among notified TB cases in migrant and local populations, Wenzhou, 2014–2023.

	MDR-TB (*n*, %)	Non MDR-TB (*n*, %)	Total	χ^2^	*P*-value
Migrant	352 (7.35%)	4,434 (92.65%)	4,786	6.250	0.012
Local	382 (6.15%)	5,825 (93.85%)	6,207		

MDR-TB, multidrug-resistant tuberculosis.

**Table 3:** Proportion of MDR-TB patients among migrant population and local residents in Wenzhou, 2014–2023.

Year	Migrant MDR-TB (*n*, %)	Local MDR-TB (*n*, %)	Total
2014	36 (40.91%)	52 (59.09%)	88
2015	47 (52.22%)	43 (47.78%)	90
2016	49 (62.82%)	29 (37.18%)	78
2017	56 (62.92%)	33 (37.08%)	89
2018	57 (60.64%)	37 (39.36%)	94
2019	62 (80.52%)	15 (19.48%)	77
2020	10 (17.54%)	47 (82.46%)	57
2021	13 (22.81%)	44 (77.19%)	57
2022	11 (25.00%)	33 (75.00%)	44
2023	11 (18.33%)	49 (81.67%)	60
χ^2^	29.842		
*P*-value	< 0.001		

MDR-TB, multidrug-resistant tuberculosis.

### 3.4 Provincial distribution of migrant MDR-TB patients

**Figure 4:**
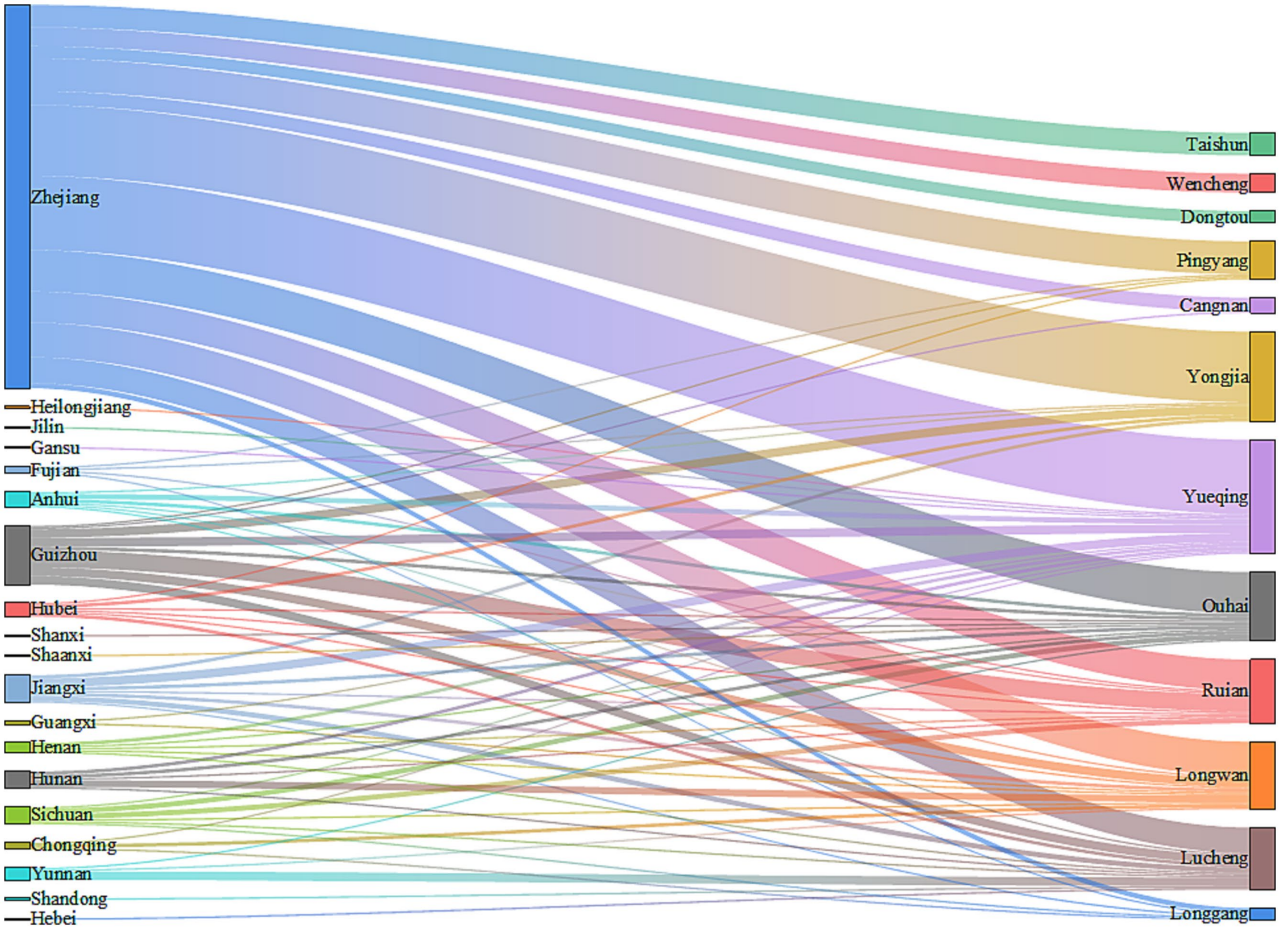
Sources and destinations of migrant MDR-TB in Wenzhou from 2014 to 2023.

Among the 352 migrant MDR-TB patients, 226 cases (64.21%) were from the migrant population within Zhejiang Province. The migrant population from outside Zhejiang Province included 37 cases (10.51%) from Guizhou Province, 18 cases (5.11%) from Jiangxi Province, 10 cases (2.84%) from Sichuan Province, and 61 cases (17.33%) from other provinces. Of the 352 migrant MDR-TB patients, 339 cases (96.31%) currently reside in Wenzhou. Among these, 222 cases (65.49%) were registered in Zhejiang Province, 34 cases (10.03%) in Guizhou Province, 16 cases (4.72%) in Jiangxi Province, 10 cases (2.95%) in Sichuan Province, 10 cases (2.95%) in Hunan Province, and 47 cases (13.86%) in other provinces (Figure 4).

### 3.5 Intra-city distribution of migrant MDR-TB patients in Wenzhou

Among the 339 migrant MDR-TB patients residing in Wenzhou, 66 cases (19.47%) were reported in Yueqing City, 52 cases (15.34%) in Yongjia County, 40 cases (11.80%) in Ouhai District, 39 cases (11.50%) in Longwan District, 37 cases (10.91%) in Rui’an City, 36 cases (10.62%) in Lucheng District, and 69 cases (20.36%) in other areas of Wenzhou City (Figure 4). The distribution of patients from Zhejiang Province predominantly occurred in Yueqing City, Yongjia County, and Ouhai District. In contrast, patients from Guizhou Province primarily migrated to Rui’an City, Lucheng District, and Longwan District. Cases from Jiangxi Province were mainly found in Yueqing City and Lucheng District, while those from Sichuan Province primarily settled in Rui’an City and Ouhai District (Figure 4). Notably, migrant MDR-TB cases in Longwan District constituted 67.24% of all MDR-TB cases, whereas Dongtou District, Ouhai District, and Taishun County accounted for 63.64, 62.50, and 56.52%, respectively (Table 4).

**Table 4:** Proportion of MDR-TB among migrant population and local residents in various districts, counties and county-level cities in Wenzhou, 2014–2023.

District/County County-level city	Migrant MDR-TB (*n*, %)	Local MDR-TB (*n*, %)	Total
Lucheng	36 (31.86%)	77 (68.14%)	113
Ouhai	40 (62.50%)	24 (37.50%)	64
Longwan	39 (67.24%)	19 (32.76%)	58
Dongtou	7 (63.64%)	4 (36.36%)	11
Cangnan	9 (23.68%)	29 (76.32%)	38
Pingyang	22 (31.43%)	48 (68.57%)	70
Yongjia	52 (54.74%)	43 (45.26%)	95
Taishun	13 (56.52%)	10 (43.48%)	23
Wencheng	11 (52.38%)	10 (47.62%)	21
Ruian	37 (41.57%)	52 (58.43%)	89
Yueqing	66 (55.93%)	52 (44.07%)	118
Longgang	7 (33.33%)	14 (66.67%)	21
Total	339 (47.02%)	382 (52.98%)	721

MDR-TB, multidrug-resistant tuberculosis.

## 4 Discussion

The results of this study indicate that the overall proportion of MDR among notified TB cases in Wenzhou City from 2014 to 2023 was 6.68%, which decreased to 5.06% in 2023. A study conducted by Yao et al. (12) in Anhui Province from 2015 to 2016 reported a MDR rate of 7.63%. Another study by Wang et al. (13) in Hainan Province from 2019 to 2021 found the MDR rate to be 19.19%. Similarly, research conducted by Wang et al. (14) in Luoyang City from 2019 to 2022 revealed a MDR rate of 11.3%. Furthermore, a study by Li et al. (15) in Hangzhou City from 2012 to 2022 reported a MDR rate of 5.3%. A study by Sambas et al. (16) in Mecca, Saudi Arabia, from 2009 to 2019 showed a MDR rate of 5.0%. A systematic review and meta-analysis of the prevalence of MDR-TB in Iran from 1981 to 2023 indicated a multidrug resistance rate of 12.31% (17). Additionally, Tengan et al. (18) systematic review and meta-analysis on the prevalence of MDR-TB in Latin America and the Caribbean reported a rate of 13.0%. A prospective cohort study by Aznar et al. (19) on the prevalence and risk factors of MDR-TB in Cubal, Angola, found a MDR rate of 25%. By comparing the results of these previous studies, we conclude that the MDR rate among TB patients in Wenzhou City is relatively low both within China and in the context of the global epidemic.

This study found that the proportion of MDR among notified patients with a history of TB treatment was higher than in new patients, which aligns with the findings of Du et al. (20) in Dalian, China, and Molla et al. (21) in East Africa. Numerous studies have identified previous treatment history as a risk factor for the development of acquired MDR (22–24). However, a study by Nsofor et al. (25) in Shanghai, China, revealed that over half of patients with a history of TB treatment developed drug resistance due to primary drug resistance rather than acquired drug resistance. Consequently, drug resistance screening should be conducted during the diagnosis and treatment of cases to facilitate the timely detection of primary multidrug resistance. Additionally, comprehensive management of the treatment of new patients should be reinforced to mitigate the risk of their progression to retreatment cases. This approach aims to reduce the spread of MDR-TB through early detection and proactive management.

From 2014 to 2023, the proportion of MDR-TB among notified cases in Wenzhou City demonstrated a significant downward trend. Concurrently, the proportions of RR-TB, HR-TB, and PDR-TB among notified cases exhibited slight decreases. These trends indicate an overall reduction in the proportion of drug-resistant TB in Wenzhou City. This positive outcome can be attributed to a series of prevention and control measures implemented over the years, including the comprehensive execution of the DOTS-Plus strategy, the expansion of pDST qualifications, and the provision of government subsidies to patients with DR-TB. Furthermore, collaborative projects, such as the prospective study on the treatment of RR-TB in partnership with the infection department team at Huashan Hospital, affiliated with Fudan University, have contributed to enhancing the success rate of MDR-TB treatment, curbing the spread of MDR-TB, and consequently reducing the drug resistance rate.

The proportion of migrant MDR-TB cases among all notified MDR-TB cases in Wenzhou City constitutes 47.96% of the total MDR-TB cases. This figure is relatively high compared to findings from other regions. For instance, a study conducted in Hangzhou indicated that migrant MDR-TB accounted for 46% of the total cases (15), while a report from Shanghai revealed that this proportion was 53% (26). Additionally, research by AI Mahrouqi et al. (27) in the Sultanate of Oman reported that migrant MDR-TB cases represented 35.6% of the total, and a study by Oliveira et al. (28) in Portugal found that the figure was 32.9%. The proportion of MDR-TB among notified migrant TB cases in this study was higher than that among notified local TB cases, aligning with results from previous studies (29, 30). The significant proportion and incidence of MDR-TB in the migrant population, corroborated by various studies indicating that migration is a risk factor for the development of MDR-TB (31, 32), underscore the necessity and importance of implementing effective prevention and control strategies specifically targeted at this demographic. Notably, the proportion of migrant MDR-TB in Wenzhou City has shown a year-on-year increase from 2014 to 2019, but declined significantly after 2020. This downward trend is consistent with our recent study in an older adult cohort [citing PMID: 40671759] (33), which employed time-series analysis to demonstrate that strict mobility-restriction policies during the COVID-19 pandemic (e.g., transportation limitations and community lockdowns) led to a transient decline in reported TB incidence during 2020–2021 (APC = −10.1, 95% CI: −15.3 to −4.7). Although the present study did not directly collect data on these control policies, the indirect evidence from the older adult-cohort study supports this inference. As we transition into the post-pandemic era, it remains imperative that prevention and control of MDR-TB among the migrant population continue to receive high priority.

The results of this study indicate that 64.21% of migrant MDR-TB patients originate from the migrant population in Zhejiang Province, while the remaining cases primarily come from areas outside Zhejiang, including Guizhou Province, Jiangxi Province, and Sichuan Province. The majority of migrant MDR-TB patients reside in Wenzhou. Our analysis identifies several contributing factors: First, Wenzhou’s economy is relatively developed, and its industrial structure is diversified (34), encompassing sectors such as manufacturing and commerce, which provide numerous employment opportunities and relatively higher income levels. This economic environment attracts labor from various levels, making it easier for the migrant population in Zhejiang Province to secure economic benefits by working and living in Wenzhou. Additionally, individuals from economically underdeveloped regions, such as Guizhou, Jiangxi, and Sichuan provinces, are drawn to Wenzhou’s economic vitality in hopes of improving their financial circumstances. Second, Zhejiang Province experiences frequent population movements, with Wenzhou, as a prominent economic hub, attracting many people from within the province. Furthermore, provinces like Guizhou, Jiangxi, and Sichuan have large population bases, contributing to significant migrant worker movements, and Wenzhou has emerged as a preferred destination for these individuals. Third, social networks formed by relatives and friends from the same village who are already residing and working in Wenzhou facilitate the migration process. Newcomers often follow these established connections, leading to a clustering effect that may result in an influx of patients into Wenzhou. These findings underscore the strong association between the migrant population and MDR-TB, highlighting the necessity for public health prevention and control strategies to account for regional economic disparities and the characteristics of the migrant population. Wenzhou has a developed economy and experiences a significant influx of population. It is advisable to enhance health screening at critical points, such as employment centers and entry points for migrant populations, to detect potential cases of MDR-TB early and prevent its spread. Additionally, it is important to increase awareness of MDR-TB in enterprises, communities, and other gathering places, thereby enhancing public understanding of prevention measures and promoting the adoption of healthy habits. Utilizing the network of local residents to train health promoters can facilitate the efficient dissemination of TB prevention and control information, establish a multi-level protection network, and ultimately reduce the risk and impact of MDR-TB transmission in Wenzhou and beyond.

Among the 339 migrant MDR-TB patients residing in Wenzhou, the primary areas of residence include Yueqing City, Yongjia County, and Ouhai District. Patients from Zhejiang Province predominantly migrate to Yueqing City, Yongjia County, and Ouhai District, while those from Guizhou Province primarily move to Rui’an City, Lucheng District, and Longwan District. Additionally, cases from Jiangxi Province mainly flow into Yueqing City and Lucheng District. Notably, migrant MDR-TB cases in Longwan District represent the highest proportion of all MDR-TB cases, accounting for 67.24%. Furthermore, Dongtou District, Ouhai District, Taishun County, Yueqing City, Yongjia County, and Lucheng District exhibit a higher prevalence of migrant MDR-TB cases, all exceeding 50%. These findings underscore the necessity of developing MDR-TB prevention and control strategies tailored for all districts and counties in Wenzhou City, providing a valuable reference for formulating effective interventions.

To translate our findings into immediate action, we propose three practical measures: Set up rapid on-site screening booths at Wenzhou South Railway Station, Shuangyu Coach Terminal, and major industrial parks; sputum smear and GeneXpert results are delivered the same day. Create a 24-h WeChat referral group linking Wenzhou with Guizhou, Jiangxi, and Sichuan to share patient information instantly and cut treatment interruption. Establish a “Migrant MDR-TB Rapid Response Team” led by Wenzhou Central Hospital together with county-level facilities to ensure referral and treatment initiation within 48 h of notification.

This study has several limitations. First, we did not assess whether these cases developed MDR-TB before or after arriving in Wenzhou, which may influence the interpretation of our results. Second, the economic structure of Wenzhou, and indeed China as a whole, is undergoing changes that could alter the composition of the migrant population in the future (35), thereby impacting the applicability of our research conclusions. Third, drug resistance was defined exclusively by phenotypic DST; the absence of genotypic data (Whole Genome Sequencing or line-probe assays) prevents us from distinguishing imported infections from local reactivation or transmission and limits insight into resistance mechanisms. Future work will integrate molecular surveillance and prospective genotypic–phenotypic linkage to refine MDR-TB control strategies.

## 5 Conclusion

Between 2014 and 2023, the proportion of MDR among notified TB cases in Wenzhou declined overall. Migrants contributed nearly half of all notified MDR-TB cases. Sustained, migrant-focused and district-specific interventions are required to further curb the spread of MDR-TB in Wenzhou.

## Data Availability

The raw data supporting the conclusions of this article will be made available by the authors, without undue reservation.
